# Neuroimmune Interactions and Their Role in Immune Cell Trafficking in Cardiovascular Diseases and Cancer

**DOI:** 10.3390/ijms26062553

**Published:** 2025-03-12

**Authors:** Yutang Wang, Jack C. Anesi, Indu S. Panicker, Darcy Cook, Prapti Bista, Yan Fang, Ernesto Oqueli

**Affiliations:** 1 Discipline of Life Science, Institute of Innovation, Science and Sustainability, Federation University Australia, Ballarat, VIC 3353, Australia; 2 Cardiology Department, Grampians Health Ballarat, Ballarat, VIC 3353, Australia; 3 School of Medicine, Faculty of Health, Deakin University, Geelong, VIC 3217, Australia

**Keywords:** noradrenaline, chemotaxis, inflammation, cardiovascular disease, cancer, inflammasome, tertiary lymphoid organ

## Abstract

Sympathetic nerves innervate bone marrow and various immune organs, where norepinephrine—the primary sympathetic neurotransmitter—directly interacts with immune cells that express adrenergic receptors. This article reviewed the key molecular pathways triggered by sympathetic activation and explored how sympathetic activity influences immune cell migration. Norepinephrine serves as a chemoattractant for monocytes, macrophages, and stem cells, promoting the migration of myeloid cells while inhibiting the migration of lymphocytes at physiological concentrations. We also examined the role of immune cell infiltration in cardiovascular diseases and cancer. Evidence suggests that sympathetic activation increases myeloid cell infiltration into target tissues across various cardiovascular diseases, including atherosclerosis, hypertension, cardiac fibrosis, cardiac hypertrophy, arrhythmia, myocardial infarction, heart failure, and stroke. Conversely, inhibiting sympathetic activity may serve as a potential therapeutic strategy to treat these conditions by reducing macrophage infiltration. Furthermore, sympathetic activation promotes macrophage accumulation in cancer tissues, mirroring its effects in cardiovascular diseases, while suppressing T lymphocyte infiltration into cancerous sites. These changes contribute to increased cancer growth and metastasis. Thus, inhibiting sympathetic activation could help to protect against cancer by enhancing T cell infiltration and reducing macrophage presence in tumors.

## 1. Introduction

The nervous and immune systems work together to defend the body against invading pathogens. Therefore, interactions between the sympathetic nervous system and immune cells play a crucial role in maintaining host health [1,2]. Research has shown that immune cells express adrenergic receptors [3], and norepinephrine—the primary sympathetic neurotransmitter—binds to these receptors to regulate immune cell function [4]. One of the key functions of immune cells is cellular trafficking, which allows them to reach target tissues.

In this review, we explored the sympathetic innervation of all lymphoid organs, including the bone marrow, thymus, spleen, lymph nodes, and tertiary lymphoid organs. We then summarized the major molecular pathways activated by sympathetic stimulation. Finally, we delved into the interactions between sympathetic activity and immune cell trafficking, discussing their implications in cardiovascular diseases and cancer, two of the leading causes of death worldwide [5,6,7].

## 2. Sympathetic Innervation of Lymphoid Organs

The lymphoid organs are categorized into primary (bone marrow and thymus) and secondary (spleen and lymph nodes) types [8,9]. Recently, tertiary lymphoid organs have also been identified [10,11,12]. This section discussed sympathetic innervation in all types of lymphoid organs.

### 2.1. Sympathetic Innervation of the Bone Marrow

**Figure 1:**
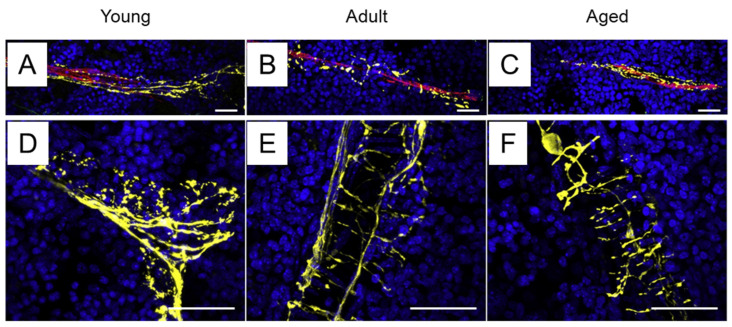
Sympathetic innervation of the bone marrow. Yellow structures represent the sympathetic nerves stained with tyrosine hydroxylase; blue structures represent nuclei stained with DAPI; and red structures represent blood vessels stained with CD31. (**A**,**D**) Young mice; (**B**,**E**) adult mice; and (**C**,**F**) aged mice. Scale bar = 30 μm. (**A**–**C**) are images with lower magnification power and (**D**–**F**) are images with higher magnification power. These images are from [16], which was published under the terms of Creative Commons CC BY 4.0 DEED (https://creativecommons.org/licenses/by/4.0/, accessed on 16 January 2024).

The bone marrow houses hematopoietic stem cells responsible for generating all immune cell lineages [13]. It is innervated by sympathetic nerves [8,14], which enter the bone through nutrient foramina and either coil around or run parallel to blood vessels in the periosteum and cortical bone [15,16,17]. Within the bone marrow, sympathetic nerves display a characteristic “corkscrew” morphology as they tightly encircle blood vessels (Figure 1). Notably, sympathetic innervation in the bone marrow declines slightly with age [16].

### 2.2. Sympathetic Innervation of the Thymus

**Figure 2:**
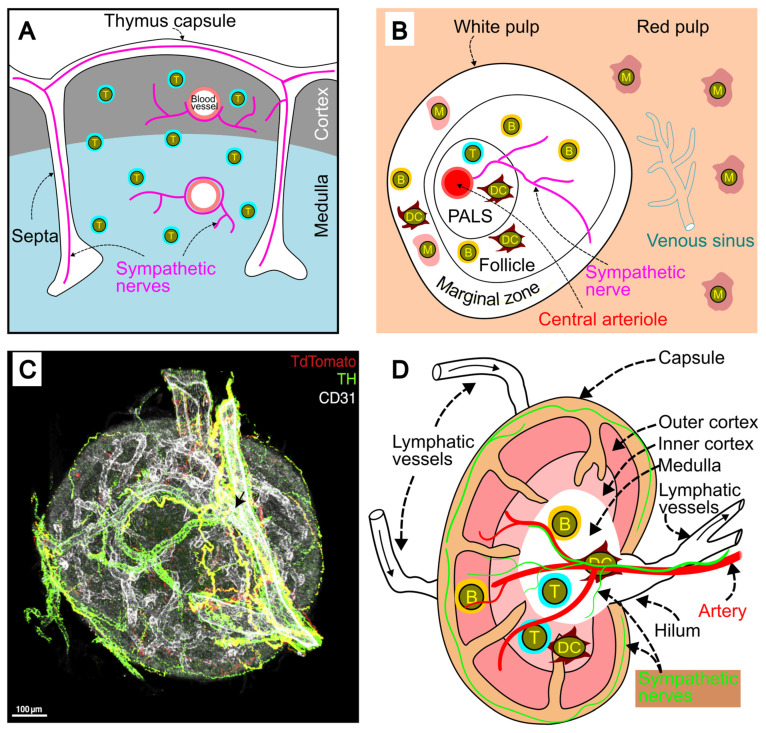
Sympathetic innervation of the thymus, spleen, and lymph nodes. (**A**) Sympathetic innervation of the thymus. Sympathetic nerves are present in the capsule, septa, cortex and medulla. These nerves also travel along blood vessels, extending into both the cortex and medulla. T, T cells. (**B**) Sympathetic innervation of the spleen. B, B cell; DC, dendritic cell; M, macrophage; PALS, periarteriolar lymphoid sheath; T, T cell. (**C**,**D**) Sympathetic innervation of the lymph node. (**C**) Three-dimensional structure of a lymph node. Green structures represent sympathetic nerves stained with tyrosine hydroxylase (TH); red structures represent sensory neurons stained with TdTomato; and white structures represent blood vessels stained with CD31. Reprinted from [21]. Copyright year, 2021, with permission from Elsevier. (**D**) Cross-sectional view of a lymph node with sympathetic nerve fibers showing in green.

The thymus is the primary site for T cell lymphopoiesis, playing a crucial role in the maturation, selection, and release of antigen-specific T cells into the periphery [18]. Sympathetic nerves are found in the capsule and interlobular septa, with some fibers penetrating the parenchyma to project into the cortex and corticomedullary junction [19] (Figure 2A). These nerves also follow blood vessels through the cortex and medulla, with some extending into both regions [19]. Sympathetic innervation of the thymus originates from the stellate ganglia and other small ganglia along the thoracic sympathetic nerve chain [20].

### 2.3. Sympathetic Innervation of the Spleen

The spleen consists of two main components: the red pulp and the white pulp [22]. The red pulp filters blood, removing damaged erythrocytes and foreign material, while the white pulp is composed of the periarteriolar lymphoid sheath (PALS), follicles, and the marginal zone (Figure 2B). As the largest secondary lymphoid organ [23], the spleen houses about one-fourth of the body’s lymphocytes and plays a critical role in immune responses to blood-borne antigens [23].

Sympathetic innervation of the spleen has been observed in various species, including humans [24], mice [25], and rats [26], and has been previously reviewed [14]. In brief, sympathetic fibers enter the spleen via the splenic nerve, with the majority of the sympathetic nerve network localized within the white pulp (Figure 2B). These nerves enter the white pulp through the central arterioles, then spread along branches of the central arteriole into the surrounding periarteriolar lymphoid sheath and follicles, where T and B cells are abundant. Occasionally, sympathetic fibers extend into the marginal zones, where macrophages, B cells, and dendritic cells reside [26,27]. Some sympathetic nerves are in close proximity to T cells, B cells, and macrophages, suggesting that they may directly influence these immune cells [27].

### 2.4. Sympathetic Innervation of Lymph Nodes

Lymph nodes are kidney-shaped organs that receive lymph through multiple afferent lymphatic vessels. The filtered lymph then exits via one or two efferent lymphatic vessels [28]. The lymph node is structured into three main regions: the capsule, cortex, and medulla (Figure 2C,D). The capsule is composed of connective tissue stroma and collagen fibers. Located beneath the capsule, the cortex consists of the outer cortex and inner cortex (or paracortex). The medulla, the innermost layer, lies at the center of the lymph node.

Lymph nodes are innervated organs [29]. Sympathetic fibers enter the lymph node via the hilum alongside blood vessels [19]. These fibers extend into the medulla and cortex, following the blood vessels (Figure 2C,D). Some sympathetic nerve fibers may project away from the vessels and extend into the cortex. Additionally, sympathetic fibers are found on the capsule surface, with some extending toward the hilum [21].

### 2.5. Sympathetic Innervation of Tertiary Lymphoid Organs

Tertiary lymphoid organs develop during persistent inflammation [30], forming unencapsulated lymphoid aggregates in chronic inflammatory diseases at locations that are often not well defined [11]. During tertiary lymphoid organ neogenesis, T and B cells, along with activated stromal lymphoid tissue organizer cells, organize themselves in or near the inflamed target tissue to form these structures [10].

**Figure 3:**
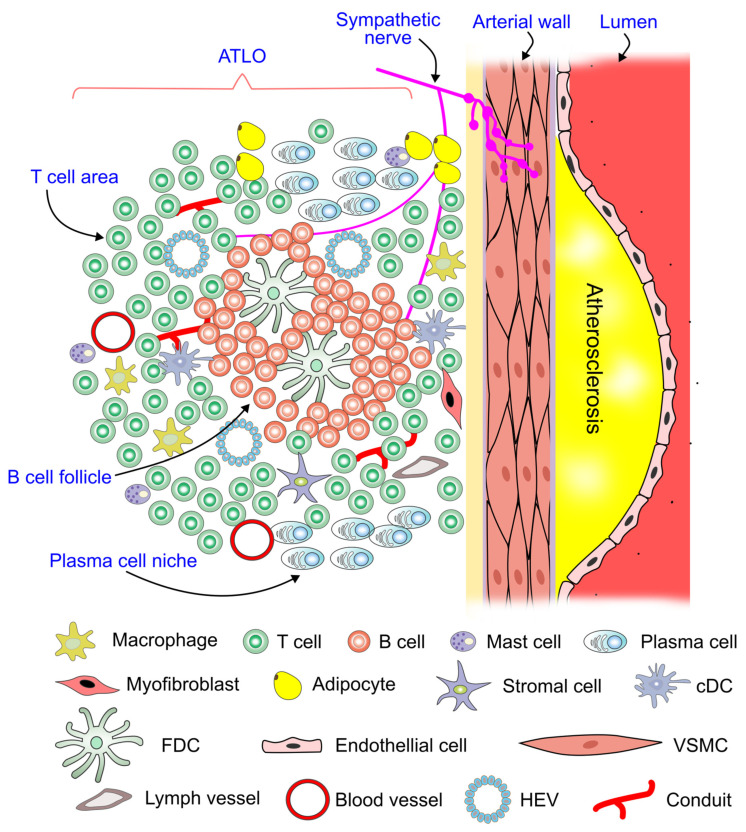
Sympathetic innervation of tertiary lymphoid organs. The shown artery tertiary lymphoid organ (ATLO) forms in response to non-resolving inflammation in atherosclerosis. Sympathetic nerves make contact with both the arterial wall and the ATLO. cDC, conventional dendritic cell; FDC, follicular dendritic cell; HEV, high endothelial venule; VSMC, vascular smooth muscle cell.

Artery tertiary lymphoid organs (ATLOs) can form adjacent to advanced atherosclerotic plaques. The cellularity, structure, and spatial organization of ATLO neogenesis reflect robust immune responses. In more advanced stages, ATLOs exhibit distinct areas for T cells, B cell follicles, and plasma cell niches at the periphery [31] (Figure 3). ATLOs are also innervated [12,32,33], with sympathetic activity playing a key role in the development of these tertiary lymphoid structures. Notably, the ablation of sympathetic innervation using 6-hydroxydopamine has been shown to impair the formation of tertiary lymphoid structures during acute lung inflammation [12].

## 3. Expression of Adrenergic Receptors on Immune Cells

There are five major types of adrenergic receptors: α1, α2, β1, β2, and β3 [3,34,35]. All of these receptors are G protein-coupled and share a common structure characterized by seven transmembrane domains [36,37]. While β3-adrenergic receptors are less well studied, they are primarily expressed in adipose tissue and the gallbladder [38,39]. β3-adrenergic receptors have also been reported in macrophages [40], neutrophils [41], and T cells [42], although their expression in lymphocytes is not consistently observed [38]. A summary of adrenergic receptor expression on immune cells is provided in Table 1. Notably, the β2-adrenergic receptor is the dominant receptor in T and B cells [42]. Additionally, the expression of adrenergic receptors on immune cells is dynamic and influenced by the surrounding environment. For instance, stress can upregulate β-adrenergic receptor expression in T cells, with a more pronounced increase in β3-adrenergic receptors [42]. Norepinephrine is more effective at activating α- and β1-adrenergic receptors compared to β2-adrenergic receptors [43,44,45]. The commonly used agonists and antagonists are listed in Table 2.

## 4. Adrenergic Signaling Pathways

Norepinephrine is the primary sympathetic neurotransmitter. Upon binding to its receptor, it activates various G proteins. α1-, α2-, and β-adrenergic receptors primarily couple with Gq/11, Gi, and Gs proteins, respectively [44,91,92], which then transmit the signal further downstream in the signaling pathway. α1-adrenergic receptors exert their effects by activating phospholipase C, which in turn increases intracellular calcium levels. In contrast, activation of α2-adrenergic receptors inhibits adenylate cyclase through Gi, while β-adrenergic receptors stimulate adenylate cyclase via Gs. Eventually, activation of adrenergic receptors can regulate the expression of inflammatory genes [93,94,95].

### 4.1. α1-Adrenergic Signaling Pathway

**Figure 4:**
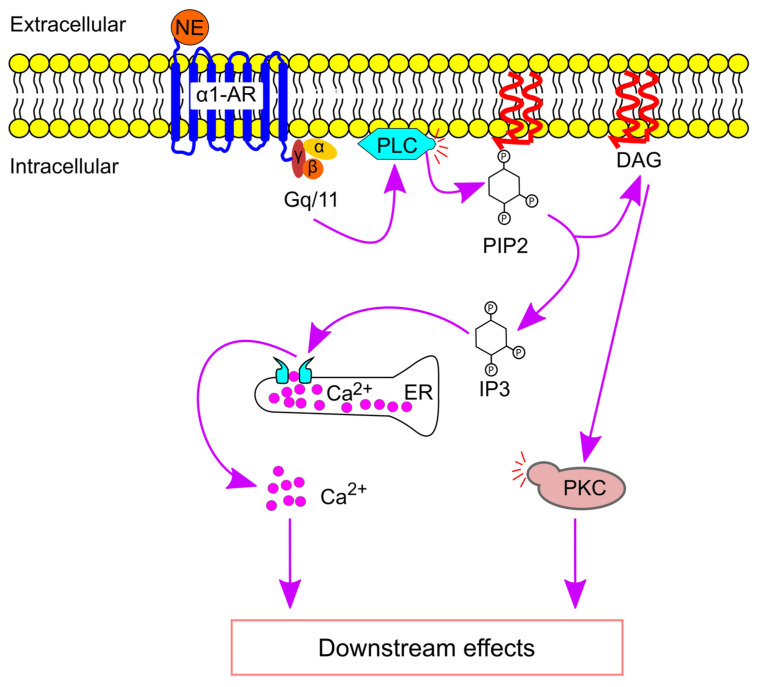
α1-adrenergic receptor (α1-AR)-mediated signaling pathway. Norepinephrine (NE) binds α1-AR, resulting in Gq/11 activation. Gq/11 further activates phospholipase C (PLC), leading to the production of inositol triphosphate (IP3) and diacylglycerol (DAG). IP3 binds to IP3 receptors on the endoplasmic reticulum (ER) to release Ca^2+^ into the cytosol. In addition, DAG activates protein kinase C (PKC), which phosphorylates downstream proteins. PIP2, phosphatidylinositol 4,5-bisphosphate.

Norepinephrine binds to the α1-adrenergic receptor, leading to the activation of Gq/11. This, in turn, activates phospholipase C, which converts phosphatidylinositol 4,5-bisphosphate into inositol 1,4,5-triphosphate (IP3) and diacylglycerol (DAG) [34]. IP3 binds to its receptor on the endoplasmic reticulum, triggering the release of stored Ca^2+^. Once in the cytosol, Ca^2+^ acts as a second messenger, mediating various downstream effects. Meanwhile, DAG activates protein kinase C, which subsequently phosphorylates downstream proteins [96] (Figure 4).

### 4.2. α2-Adrenergic Signaling Pathway

**Figure 5:**
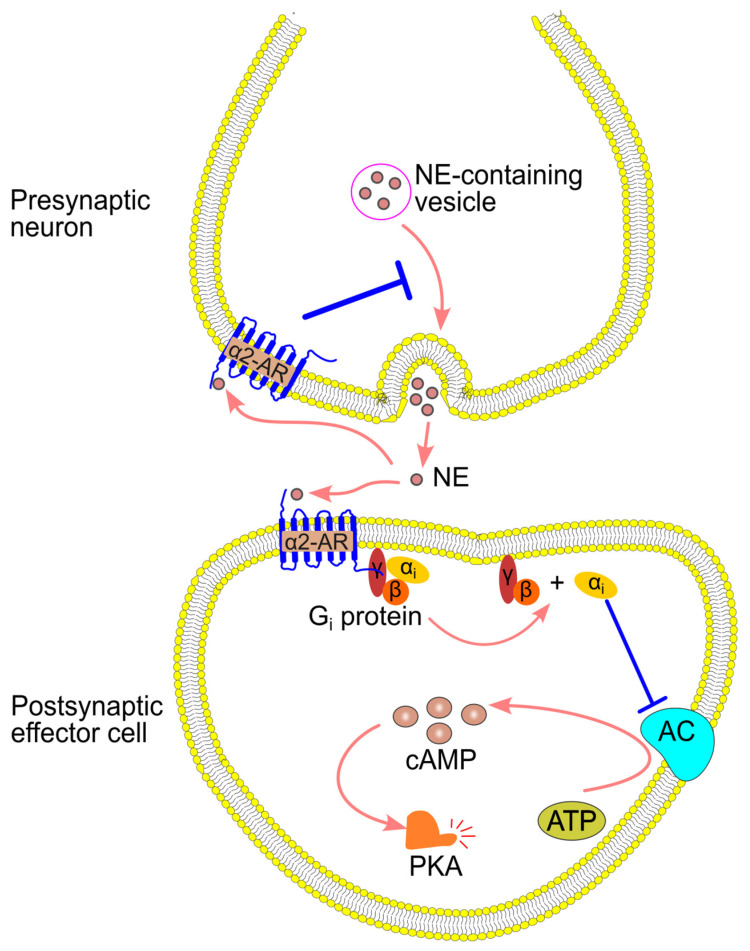
α2-adrenergic receptor (α2-AR)-mediated signaling pathway. Norepinephrine (NE) binds α2-AR, resulting in Gi activation, which then inhibits adenylyl cyclase (AC). In addition, α2-AR in the presynaptic membrane inhibits NE release. ATP, adenosine triphosphate; cAMP, cyclic adenosine 3′,5′-monophosphate; PKA, protein kinase A.

α2-adrenergic receptors have both presynaptic and postsynaptic roles. On presynaptic neurons, α2-adrenergic receptors regulate norepinephrine release through a negative feedback mechanism, reducing further neurotransmitter release. On postsynaptic cells, these receptors couple to Gi proteins, which inhibit adenylyl cyclase and decrease the production of cyclic adenosine 3′,5′-monophosphate (cAMP) [91,97,98,99,100,101] (Figure 5).

### 4.3. β-Adrenergic Signaling Pathway

**Figure 6:**
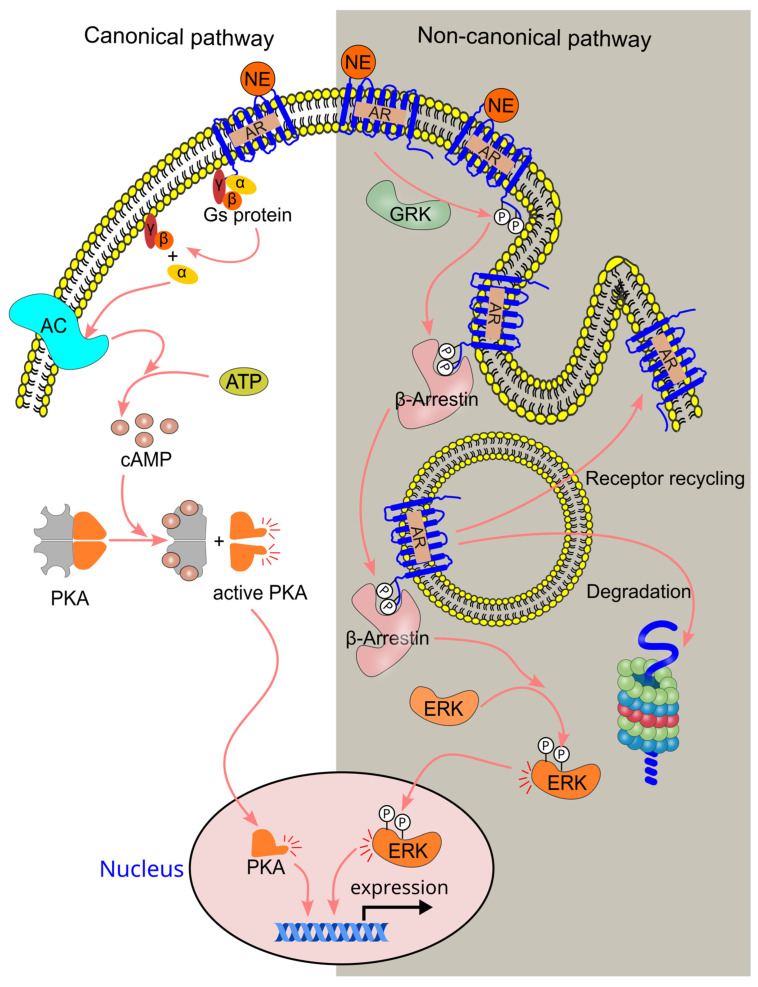
β-adrenergic receptor-mediated signaling pathways. The canonical pathway is G-protein-dependent (left side), while the non-canonical pathway is G-protein-independent (right side). AC, adenylyl cyclase; ATP, adenosine triphosphate; cAMP, cyclic adenosine 3′,5′-monophosphate; ERK, extracellular signal-regulated kinase; GRK, G-protein-coupled receptor kinase; NE, norepinephrine; PKA, protein kinase A.

β-adrenergic receptors can signal through both canonical and non-canonical pathways. The canonical pathway, which is G-protein-dependent, and the non-canonical pathway, which is G-protein-independent, mediate distinct cellular responses [3] (Figure 6). β2-adrenergic receptors are particularly abundant in neuroimmune communication [3].

In the canonical pathway, epinephrine binds to β-adrenergic receptors, causing a conformational change that activates the Gs protein through the dissociation of the α subunit from the βϒ subunits. The activated α subunit then stimulates adenylyl cyclase, a membrane-bound enzyme, which converts ATP to cAMP [102,103]. cAMP binds to the regulatory subunits of protein kinase A (PKA), which exists as a holoenzyme composed of two regulatory subunits and two catalytic subunits [104]. This binding induces the dissociation of the PKA holoenzyme, releasing the free catalytic subunits [105], which then phosphorylate downstream molecules. One such molecule is the cAMP response element-binding protein (CREB), a transcription factor. Phosphorylated CREB recruits the coactivator CREB-binding protein, stimulating the transcription of target genes (Figure 6).

In the non-canonical pathway, G-protein-coupled receptor kinase (GRK) phosphorylates β-adrenergic receptors [106], creating a binding site for β-arrestin [107]. The binding of β-arrestin leads to the internalization (endocytosis) of the adrenergic receptor [108], which may either be recycled back to the cell surface or degraded in the lysosome. β-arrestins can also function as signal transducers, interacting with various signaling molecules in the cytoplasm and initiating downstream signaling [109]. For instance, β-arrestins can activate ERK and regulate gene expression [110].

The canonical pathway can be regulated by the non-canonical pathway. When the canonical pathway is activated by a high concentration of norepinephrine, PKA can phosphorylate GRK, enhancing GRK-mediated phosphorylation of β-adrenergic receptors and triggering the activation of the non-canonical pathway [106]. This results in the binding of β-arrestin, which inhibits β-adrenergic receptor activity. Additionally, β-arrestin binding recruits and activates phosphodiesterase, which hydrolyzes cAMP, effectively inactivating the canonical cAMP signaling pathway [111,112].

## 5. Normal Range of Circulating Norepinephrine

The normal range of circulating norepinephrine is 0.4–10.0 nM [113], though this can vary slightly between laboratories. Norepinephrine concentrations fluctuate depending on the body’s condition. For instance, it doubles when a person transitions from a supine to an upright position [114] and increases during exercise [115,116]. Norepinephrine levels can reach 1 μM inside the tissue near nerve fibers [74,117]. Research has shown that norepinephrine is not cytotoxic to neutrophils at concentrations up to 10 μM [1], but it can be cytotoxic to macrophages at doses above 3 μM [118].

## 6. Effect of Sympathetic Activation on Circulating Leukocyte Numbers

It has been demonstrated that norepinephrine injection in humans leads to a rapid, transient increase in circulating leukocytes [119,120]. This section discussed the effect of norepinephrine on various circulating leukocyte numbers.

### 6.1. Effect of Norepinephrine on Numbers of Circulating Innate Immune Cells

Intramuscular administration of norepinephrine resulted in a significant increase in circulating neutrophil numbers 15 min after injection [119]. When administered intravenously, norepinephrine elevated neutrophil counts during the infusion, immediately after the infusion, and 30 min post-infusion [121]. Interestingly, neither the β2-adrenergic receptor agonist salbutamol nor the non-selective β-adrenergic receptor agonist isoprenaline increased neutrophil counts, suggesting that β-adrenergic receptors may not be involved in the norepinephrine-induced rise in circulating neutrophils. However, β1-adrenergic receptors could play a role in the acute stress-induced increase in neutrophil circulation. For example, acute cold restraint (restraining mice in a well-ventilated 60 mL syringe at 4 °C for 1 h) led to an increase in circulating neutrophils, which was mediated by β1-adrenergic receptors, promoting the release of neutrophils from the bone marrow [41].

Intramuscular administration of norepinephrine in humans resulted in an increase in circulating eosinophils 15 min after injection [119,122], with eosinophil levels returning to baseline within 60 min [119]. The spleen plays a role in mediating the norepinephrine-induced rise in circulating eosinophils [119].

Administration of norepinephrine (either via subcutaneous injection at a dose of 10 μg/kg [120] or intravenous injection at 0.15 μg/kg/min for 20 min [123]) to healthy subjects resulted in a rapid increase in plasma natural killer (NK) cell numbers, which returned to baseline within 60 min after administration [120,123]. This norepinephrine-induced increase in NK cell numbers was also observed in splenectomized subjects, indicating that NK cells are recruited from sources other than the spleen [123]. This notion is further supported by the finding that acute psychological stress (such as a parachute jump) increased NK cell numbers in splenectomized subjects to levels comparable to those in individuals with intact spleens [124]. The β1-selective antagonist bisoprolol did not inhibit the norepinephrine-induced increase in NK cell numbers, while the non-selective β-adrenergic receptor antagonist propranolol did, suggesting that norepinephrine increases circulating NK cells via β2-adrenergic receptors in a spleen-independent manner. Consistently, β2-adrenergic receptors mediate the exercise-induced increase in circulating NK cells [84].

### 6.2. Effect of Norepinephrine on Circulating Lymphocyte Numbers

Administration of norepinephrine to healthy subjects via intravenous infusion led to an increase in circulating lymphocytes (lymphocytosis) [121], a finding confirmed by other studies [119] (Table 3). The spleen plays a role in this increase, as a preclinical study demonstrated that norepinephrine (150 μg/kg, intracardiac injection) enhanced lymphocyte release from the spleen in guinea pigs [125]. Additionally, phentolamine, an α-adrenergic receptor antagonist, blocked the norepinephrine-induced increase in lymphocyte release from the spleen [125], suggesting that α-adrenergic receptors mediate norepinephrine-induced lymphocytosis.

β2-adrenergic receptors may also contribute to norepinephrine-induced lymphocytosis. The β2-selective agonist salbutamol increased circulating lymphocyte counts [121], likely through the release of lymphocytes from the spleen [83]. Furthermore, β2-adrenergic receptors mediate the exercise-induced increase in overall circulating lymphocytes and CD8^+^ T cells [84]. In human lymphocytes, the increase in cAMP induced by isoproterenol was inhibited by the β2-selective antagonist ICI 118,551, but not by the β1-selective antagonist bisoprolol [83], indicating that β2 is the primary β signaling pathway in lymphocytes. Thus, both α- and β2-adrenergic receptors may contribute to norepinephrine-induced lymphocytosis.

Norepinephrine-induced lymphocytosis is primarily driven by an increase in T cell numbers. It has been shown that norepinephrine did not affect circulating B cell levels [83,123]. In contrast, norepinephrine increased the number of circulating total T cells (CD3^+^), as well as CD4^+^ and CD8^+^ T cells, during and immediately after infusion [123] (Table 3). However, this increase in T cell numbers is transient, as circulating T cell counts returned to baseline within 30 min after norepinephrine infusion [123], explaining why no effect on circulating T cell numbers is observed when blood is collected ≥30 min after infusion [83,121] (Table 3). The transient nature of the norepinephrine-induced increase in T cell numbers may also depend on the route of administration, as subcutaneous administration of norepinephrine did not increase circulating T cell numbers [120] (Table 3).

## 7. Norepinephrine Is a Chemoattractant for Monocytes, Macrophages, Stem, and Progenitor Cells

Norepinephrine acts as a chemoattractant for monocytes, macrophages, and CD34^+^ stem and progenitor cells [126,127,128] (Table 4). The norepinephrine-induced chemotaxis of human monocytes and macrophages is mediated through β-adrenergic receptors, which activate adenylyl cyclase, leading to an increase in cAMP [126]. Additionally, in human monocytic THP-1 cells transfected with α2A-adrenergic receptors, the α2-adrenergic receptor agonist UK 14,304 induced chemotaxis of these transfected cells [129], suggesting that α2-adrenergic receptors may also contribute to norepinephrine-induced chemotaxis. However, α2-adrenergic receptors likely do not play a major role in human monocyte chemotaxis under physiological conditions, as activation of either α1 or α2-adrenergic receptors did not induce chemotaxis in isolated human monocytes [126].

The chemotactic effect of norepinephrine on monocytes and macrophages appears to be limited to lower concentrations (10^−11^ to 10^−8^ M), as higher concentrations (3 × 10^−7^ and 6 × 10^−7^ M) did not elicit a chemotactic response [127] (Table 4).

## 8. Effect of Sympathetic Activity on Immune Cell Trafficking

### 8.1. Effect of Sympathetic Activity on Monocyte and Macrophage Trafficking

Norepinephrine at lower concentrations (10^−12^ to 10^−8^ M) has been shown to enhance fMLP-induced migration of macrophages [130,131], with α-adrenergic receptors playing a key role in this process, particularly in mouse macrophages [130] (Table 5).

It is important to note that the effect of norepinephrine on migration is concentration-dependent. While lower concentrations (10^−12^ to 10^−8^ M, within the normal circulating range) promote macrophage migration, higher concentrations (10^−8^ to 10^−5^ M) do not have the same effect [127,130,131] (Table 5). Additionally, the impact of norepinephrine on macrophage migration is age-dependent. For instance, lower concentrations (10^−12^ M) enhance fMLP-induced migration of macrophages in 12- and 22-week-old mice, but not in mice aged 72 weeks [131]. Furthermore, higher concentrations (10^−5^ M) actually reduced macrophage migration in older mice [131]. This inhibitory effect on migration may be attributed to the cytotoxicity of norepinephrine, as a previous study reported that it became cytotoxic to macrophages at concentrations exceeding 3 μM [118]. In certain stress conditions, norepinephrine can also inhibit macrophage migration, as shown by the reduction in migration of AlCl_3_-stressed macrophages at 1 and 10 nM norepinephrine [62].

### 8.2. Effect of Sympathetic Activity on Neutrophil Trafficking

Lower doses of norepinephrine within the physiological range (e.g., 4 nM) have been shown to promote human neutrophil migration [132] (Table 6). The molecular mechanisms underlying this effect remain unclear, but it may be mediated by the α1-adrenergic receptor, as stimulation of this receptor with phenylephrine has been shown to enhance neutrophil migration toward a bacterial chemotactic factor [133]. Additionally, α1-, α2-, β1-, and β2-adrenergic receptors have all been implicated in the increase in human neutrophil migration induced by extracellular heat shock proteins [134]. These findings suggest that both α- and β-adrenergic receptors may play a role in norepinephrine-induced neutrophil migration.

In contrast, higher concentrations of norepinephrine (10^−7^ to 10^−3^ M) have been shown to decrease neutrophil migration [1,133,135] (Table 6). For instance, norepinephrine (10^−5^ M) superfusion inhibited f-Met-Leu-Phe peptide (fMLP)-induced neutrophil transmigration in vivo [1]. The reduction in migration at high norepinephrine concentrations may be mediated by the activation of β-adrenergic receptors and an increase in cAMP, as isoproterenol, a non-selective β-adrenergic receptor agonist, decreased neutrophil migration through a similar cAMP-mediated mechanism [133].

### 8.3. Effect of Sympathetic Activity on NK Cell Trafficking

NK cells are innate lymphocytes that play a crucial role in killing virally infected, stressed, or cancerous cells [136]. These cells express germline-encoded receptors and do not undergo antigen receptor rearrangement, classifying them as part of the innate immune system [137,138].

Sympathetic signaling influences NK cell trafficking. For example, norepinephrine (10^−6^ M) has been shown to enhance the migration of NK cells from human peripheral blood [72]. Similarly, psychological stress, such as a public speaking task, increases circulating NK cell numbers, a response that is linked to elevated circulating norepinephrine levels [139].

### 8.4. Effect of Sympathetic Activity on Lymphocyte Trafficking

Some studies have shown that norepinephrine does not affect CD8^+^ T cell migration induced by chemoattractants [54,72,77] (Table 7). However, other research indicates that norepinephrine inhibits lymphocyte migration [55,77,81], with both α- and β-adrenergic receptors playing a role in this inhibition [55,77,81]. Additionally, norepinephrine has been found to reduce lymphocyte migration by impacting other cell types. For example, Geng et al. demonstrated that norepinephrine administration (2 mg/mg) decreased CD8^+^ T cell infiltration into tumor tissue in C57BL/6 mice [77]. This effect was mediated through β2-adrenergic receptor signaling, which reduced C-X-C motif chemokine ligand 9 (CXCL9) secretion by tumor cells, thereby limiting CD8^+^ T cell infiltration into the tumor [77].

### 8.5. Effect of Sympathetic Activity on Immune Cell Splenic Retention

Mice deficient in β2-adrenergic receptors in the bone marrow exhibited larger spleens and increased retention of monocytes, macrophages, mast cells, and neutrophils in the spleen [87]. This increased splenic retention of leukocytes was associated with elevated VCAM-1 expression in the spleen [87]. Pharmacological inhibition of β2-adrenergic receptors, but not β1-adrenergic receptors, also resulted in enlarged spleens and increased VCAM-1 expression in mice [86]. Consistent with these findings, spleens from human tissue donors treated with β-adrenergic receptor blockers showed higher VCAM-1 expression [87]. Additionally, selective inhibition of β2-adrenergic receptors promoted the accumulation of monocytes, macrophages, mast cells, and neutrophils in the red pulp of the spleen in mice [86].

**Figure 7:**
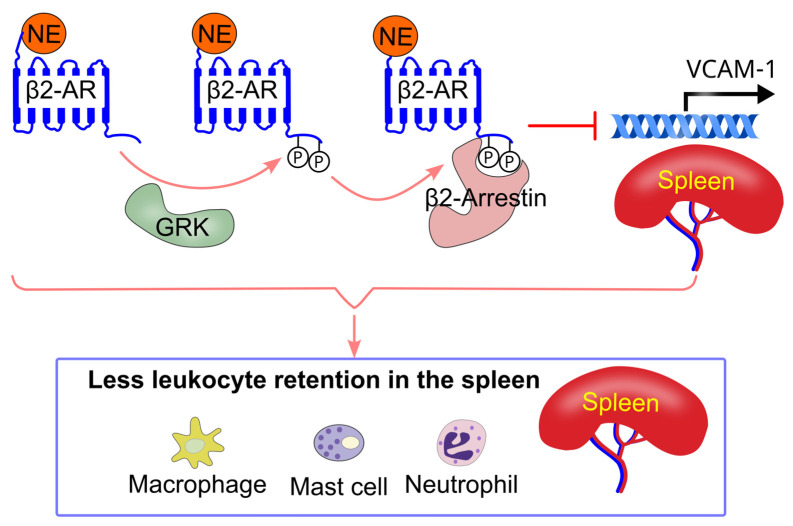
β2 activation reduces leukocyte retention in the spleen. AR, adrenergic receptor; GRK, G-protein-coupled receptor kinase; VCAM-1, vascular cell adhesion molecule 1.

Treatment with the β2-adrenergic receptor-selective agonist salbutamol in mice reduced VCAM-1 expression in bone marrow-derived macrophages. Similarly, salbutamol treatment decreased VCAM-1 expression in a human macrophage cell line [87]. Genetically, lentivirus-mediated restoration of β2-adrenergic receptor expression in β2-adrenergic receptor knockout macrophages led to a reduction in VCAM-1 expression [87]. Mechanistically, β2-adrenergic receptor inhibition-induced VCAM-1 expression is mediated via β2-arrestin-dependent signaling [87] (Figure 7).

### 8.6. Effect of Sympathetic Activity on Bone Marrow Cell Migration

Sympathetic activation has been shown to promote myelopoiesis [140,141,142]. For instance, stress-induced sympathetic activation in mice (e.g., burn sepsis or repeated social defeat) led to an increase in monocytes and granulocytes in the bone marrow, spleen, and peripheral blood, accompanied by an overall enhancement of myelopoiesis [140,141,142]. Inhibition of sympathetic activity, achieved through 6-hydroxydopamine or the β-adrenergic receptor antagonist propranolol, suppressed myelopoiesis [140,141]. Similarly, in humans, social stress (e.g., low socioeconomic status) was associated with elevated circulating monocytes [142]. These findings suggest that sympathetic activation drives myelopoiesis via β-adrenergic receptors.

The molecular events downstream of sympathetic activation that contribute to myelopoiesis may involve an increase in cAMP, activation of cAMP response element-binding protein (CREB), and elevated expression of GM-CSF [142]. Notably, sympathetic activation does not appear to promote lymphopoiesis, as stress has been shown to decrease lymphoid progenitors in the bone marrow [142].

**Figure 8:**
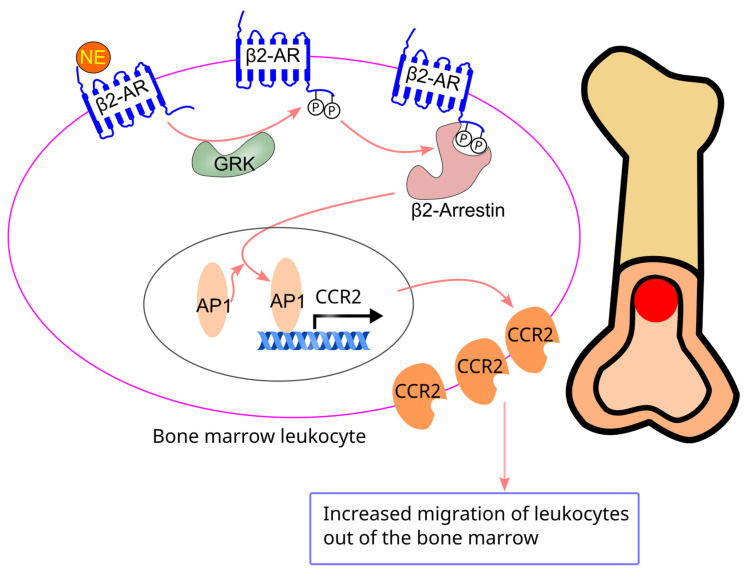
β2-adrenergic receptors promote migration of bone marrow leukocytes. AP1, activator protein-1; AR, adrenergic receptor; GRK, G-protein-coupled receptor kinase; CCR2, C-C chemokine receptor 2.

Bone marrow cells from β2-adrenergic receptor knockout mice exhibited reduced CCR2 expression and impaired migration toward C-C motif chemokine ligand 2 (CCL2) [75]. Lentiviral-mediated restoration of β2-adrenergic receptors in these cells restored CCR2 expression and migration toward CCL2 [75]. Consistently, treatment with the β2-adrenergic receptor-selective agonist salbutamol increased CCR2 expression in the bone marrow and enhanced migration of bone marrow cells toward CCL2. The β2-adrenergic receptor-induced increase in CCR2 expression and migration is mediated through the GRK-β2-arrestin pathway, which activates activator protein-1 (AP1), leading to increased AP1 binding to the CCR2 promoter [75] (Figure 8). In line with this, chronic treatment with β2-adrenergic receptor blockers decreased both CCR2 expression and migration of bone marrow cells in mice [86].

Norepinephrine may also facilitate the egress of stem cells to their target tissues. The granulocyte colony-stimulating factor (G-CSF)-induced mobilization of hematopoietic stem and progenitor cells (HSPCs) from the bone marrow to circulation is mediated by sympathetic activity. Pharmacological inhibition of sympathetic activity with 6-hydroxydopamine or β-adrenergic receptor blockers, such as propranolol, decreased G-CSF-induced egress of HSPCs from the bone marrow [76]. Furthermore, in mice deficient in dopamine β-hydroxylase (the enzyme responsible for converting dopamine to norepinephrine [34]), G-CSF-induced mobilization of HSPCs was impaired. However, treatment with the β2-adrenergic receptor agonist clenbuterol partially rescued this mobilization defect, suggesting that β2-adrenergic receptors play a crucial role in stem cell mobilization. Additionally, norepinephrine (10^−6^ M) treatment of CD34^+^ stem and progenitor cells increased membrane type-1 matrix metalloproteinase (MT1-MMP) expression and MMP-2 activity [128], which may help facilitate HSPC mobilization and egress [143]. Norepinephrine treatment also enhanced the engraftment of CD34^+^ cells into the bone marrow following sublethal irradiation, suggesting that norepinephrine promotes stem cell migration (homing) [128].

### 8.7. Effect of Sympathetic Activity on Peripheral Blood Leukocyte Migration

Chronic treatment with β2-adrenergic receptor blockers has been shown to reduce CCR2 expression and impair the migration of peripheral blood leukocytes in both mice and humans [86]. These findings suggest that sympathetic activation enhances the migration of peripheral blood leukocytes through β2-adrenergic receptors.

### 8.8. Effect of Sympathetic Activity on Motility of Lymph Node Lymphocytes

Norepinephrine (10 μM) superfusion has been shown to decrease T and B cell movement within lymph nodes in mice, with the effect being both rapid and reversible [74]. Interestingly, chemical sympathectomy with 6-hydroxydopamine (6-OHDA) did not affect T cell motility within the lymph nodes [74], suggesting that while physiological sympathetic activity may not significantly influence T cell motility, high doses of norepinephrine can inhibit it.

The inhibitory effect of norepinephrine on lymphocyte movement within the lymph nodes is mediated by α1-, α2-, and β2-adrenergic receptors expressed on non-hematopoietic cells in the tissue [74]. Mechanistically, norepinephrine activates these receptors, which leads to the constriction of microvasculature within the lymph nodes, inducing hypoxia. This hypoxic environment then triggers an increase in intracellular Ca^2+^ concentration in lymphocytes, ultimately resulting in a halt to their motility [74].

Lymphocyte migration from lymph nodes to the blood follows a circadian rhythm, increasing when sympathetic activity decreases and vice versa when sympathetic activity rises. This migration process is regulated by β2-adrenergic receptors on lymphocytes [144]. During the active phase, when sympathetic activity is higher, more lymphocytes remain in the lymph nodes. This migration-inhibitory effect of sympathetic activity in the normal circadian rhythm appears to be beneficial, as it can enhance B lymphocyte function, including increased antibody production [144].

Activation of adrenergic receptors by β2 agonists does not affect lymphocyte entry into the lymph nodes [74]. However, the reduced motility of lymphocytes within the lymph nodes may hinder their ability to encounter antigen-presenting dendritic cells, ultimately impairing their capacity to kill virus-infected cells [74].

### 8.9. Effect of Sympathetic Activity on Immune Cell Interaction with Endothelial Cells

The mechanism underlying leukocyte migration across the endothelium is well-characterized and has been described in detail elsewhere [145,146,147]. This process involves several key steps, including rolling, slow rolling, arrest, adhesion strengthening, intravascular crawling, and transmigration [148,149,150]. These steps are regulated jointly by endothelial cells and circulating leukocytes [148,149,150,151].

Norepinephrine can promote the adhesion of neutrophils and monocytes to endothelial cells. For instance, it has been shown that norepinephrine (1 µM) increased the expression of ICAM-1 (intercellular adhesion molecule 1) and VCAM-1 (vascular cell adhesion molecule 1) in endothelial cells [152]. Additionally, norepinephrine enhanced the secretion of CCL7 by endothelial cells via α-adrenergic receptors [152]. As a result, the priming of endothelial cells with norepinephrine led to an increased adhesion of neutrophils and monocytes to these cells [152].

The increase in neutrophil and monocyte adhesion to endothelial cells induced by norepinephrine is mediated through α-adrenergic receptors, which subsequently promote the secretion of CCL7 by cultured endothelial cells [152]. However, some studies report contradictory findings. For example, superfusion of mice with a high concentration of norepinephrine (10 µM) inhibited fMLP-induced neutrophil adhesion in vivo [1]. Furthermore, norepinephrine at this concentration reduced the adhesion of human neutrophils to cultured endothelial cells by decreasing β2-integrin expression [54].

In contrast, norepinephrine significantly increases the adhesion and rolling of activated CD8^+^ T cells [54]. It also significantly elevates interleukin-8 (IL-8) release from endothelial cells, with an associated increase in the IL-8 receptor (CXCR1) on activated CD8^+^ T cells [54,55]. Blocking the CXCR1 receptor with an antibody reduced the adhesion of activated CD8+ T lymphocytes to endothelial cells in the presence of norepinephrine. These findings suggest that norepinephrine-mediated IL-8 release from endothelial cells plays a key role in the migration of activated CD8^+^ T cells to sites of inflammation, highlighting norepinephrine’s role in fine-tuning T cell responses. However, norepinephrine (≥1 µM) did not affect the rolling or adhesion of human naïve CD8^+^ cells to endothelial cells [55].

## 9. Sympathetic Activation and Immune Cell Trafficking in Cardiovascular Diseases (CVDs)

CVDs encompass a range of disorders affecting the heart and blood vessels [153,154,155], and they represent the leading cause of death worldwide, responsible for approximately 17.9 million deaths annually [5,6]. Atherosclerosis, the primary underlying cause of CVDs, plays a central role in the development of these conditions. Behavioral risk factors, such as an unhealthy diet [156,157], physical inactivity [158], tobacco use [159], and excessive alcohol consumption [160], can worsen atherosclerosis and increase the risk of CVDs. Additionally, hypertension [161,162], diabetes [163,164], and elevated blood lipids [165,166] are significant risk factors. Effective management of these conditions can reduce the risk of CVDs and help prevent heart attacks and strokes [167].

CVDs are marked by inflammation, with atherosclerosis—the primary underlying cause—being recognized as a chronic inflammatory condition [168]. During this process, inflammatory cells such as macrophages accumulate, contributing to plaque formation [169]. Over time, this plaque can rupture, potentially triggering the formation of blood clots and leading to serious cardiovascular events, including heart attacks and strokes [170].

### 9.1. Sympathetic Activation and Immune Cell Trafficking in Atherosclerosis

Atherosclerosis is characterized by the gradual buildup of plaque inside the arteries [171], leading to their hardening and restricting blood flow over time [172]. The artery is an innervated organ [34], with the adventitia serving as the primary conduit for the nervous system to reach peripheral tissues [173,174]. Sympathetic activity plays a crucial role in the pathogenesis of atherosclerosis [175].

**Figure 9:**
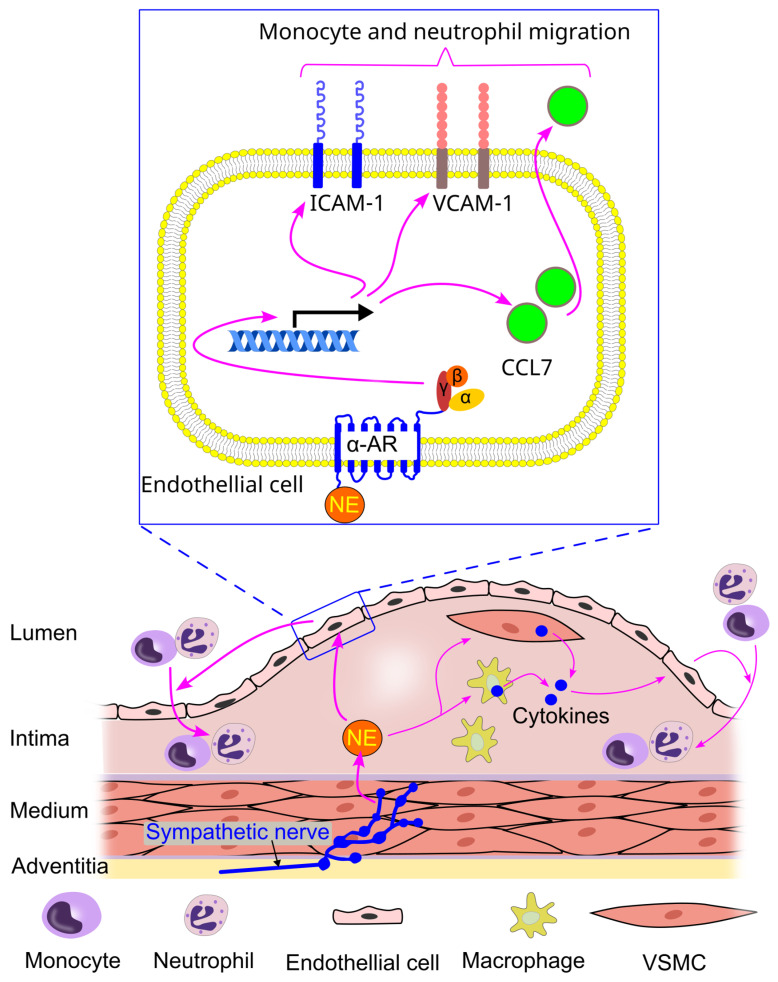
Sympathetic activation-induced immune cell infiltration in atherosclerosis. Locally produced norepinephrine activates endothelial cells via α-adrenergic receptors, leading to increased expression of adhesion molecules (e.g., ICAM-1 and VCAM-1) and chemoattractant molecules (e.g., CCL7). This promotes the adhesion and infiltration of myeloid cells. Additionally, norepinephrine stimulates the release of proinflammatory cytokines (e.g., IL-1 and IL-6) from macrophages and vascular smooth muscle cells (VSMCs), which may further activate endothelial cells and enhance myeloid cell infiltration. AR, adrenergic receptor; CCL7, C-C motif chemokine ligand 7; ICAM-1, intercellular adhesion molecule 1; IL, interleukin; VCAM-1, vascular cell adhesion molecule 1.

Hinterdobler et al. reported that mental stress elevated norepinephrine levels in the blood vessel walls, which in turn promoted the migration of myeloid cells (monocytes and neutrophils) into atherosclerotic lesions and increased the incidence of plaque rupture in mice [152]. This increased myeloid cell migration was associated with elevated protein levels of ICAM-1 and VCAM-1 on the endothelial cells of the aorta, as well as heightened chemokine production (e.g., CCL7) by these endothelial cells [152]. In vitro experiments further demonstrated that norepinephrine treatment enhanced leukocyte adhesion to endothelial cells, a process mediated by α-adrenergic receptors and subsequent CCL7 secretion by cultured endothelial cells [152] (Figure 9).

Inhibition of sympathetic activity through 6-hydroxydopamine or surgical denervation reduced neutrophil and monocyte migration into atherosclerotic lesions, which was linked to a suppression of stress-induced chemokine production (e.g., CCL7) [152]. In contrast, bilateral adrenalectomy (removal of the adrenal glands) did not have the same effect [152], suggesting that locally produced norepinephrine, rather than systemically sourced norepinephrine, plays a key role in promoting stress-induced leukocyte migration into the plaque.

Moreover, norepinephrine can stimulate the release of proinflammatory cytokines (e.g., IL-1 and IL-6) from macrophages and vascular smooth muscle cells [152]. These cytokines may further contribute to endothelial activation, promoting the migration of myeloid cells into atherosclerotic lesions (Figure 9).

In atherosclerosis, the persistent inflammation triggers sympathetic axon neogenesis in the adventitia, leading to the formation of artery tertiary lymphoid organs (ATLOs) [33]. This axon growth is confined to atherosclerotic regions throughout the major arterial tree [33]. The development of ATLOs correlates with the size of atherosclerotic lesions, as well as plaque instability and rupture in human coronary arteries [176]. In mice, inhibition of sympathetic nerve activity using 6-hydroxydopamine reduced aortic norepinephrine levels, diminished the number and size of ATLOs [33], suppressed T and B cell infiltration, and completely eliminated ATLO structures [33]. Furthermore, surgical sympathetic denervation reduced the number of CD11b^+^ myeloid cells in the spleen, decreased ATLO size and quantity, and attenuated atherosclerosis [33].

### 9.2. Sympathetic Activation and Immune Cell Trafficking in Hypertension

Hypertension is a risk factor for atherosclerosis and CVDs [177,178,179]. Immune cells play a crucial role in the pathogenesis of hypertension [180,181]. For instance, Guzik et al. demonstrated that mice deficient in T cells were resistant to hypertension induced by angiotensin II or deoxycorticosterone acetate [182]. In contrast, adoptive transfer of T cells restored the hypertensive response [182].

The sympathetic nervous system is also activated in hypertension [183]. Numerous studies have shown that sympathetic activation in the context of hypertension leads to immune cell infiltration into the vessel walls, kidneys, and central nervous system [66,184,185,186]. This infiltration plays a pivotal role in the development of hypertension [180,187].

Sympathetic activation can drive immune cell infiltration into the aorta. For example, Michell et al. reported that norepinephrine infusion in mice increased blood pressure, accompanied by elevated aortic ICAM-1 expression and enhanced leukocyte adhesion to the aorta [184]. Similarly, Xiao et al. found that angiotensin II infusion resulted in hypertension and increased infiltration of leukocytes (including CD4^+^ and CD8^+^ T cells) into the aorta. Notably, renal sympathetic denervation, an antihypertensive treatment [188], attenuated both hypertension and leukocyte infiltration [66].

Sympathetic activation also leads to immune cell infiltration into the kidneys. For instance, norepinephrine infusion increased renal ICAM-1 expression and macrophage infiltration [184]. This macrophage infiltration was mediated by mechanosensor caveolin-1 in endothelial cells and the subsequent production of ROS [184]. Increased immune cell infiltration into the kidneys has been reported in various animal models of hypertension. For example, macrophage infiltration into the renal medulla was heightened in nicotine-induced hypertension [189]; T cell infiltration increased in deoxycorticosterone acetate-induced hypertension [185]; and in angiotensin II-induced hypertension, there was an increased infiltration of total leukocytes, monocytes, macrophages, and T cells (both CD4^+^ and CD8^+^) into the kidneys [66]. The angiotensin II-induced rise in immune cell infiltration was facilitated by local increases in adhesion and chemoattractant molecules (VCAM-1, ICAM-1, MCP-1 [monocyte chemoattractant protein-1], and RANTES [regulated on activation, normal T cell expressed and secreted]), and this effect was blocked by renal denervation [66]. Norepinephrine can also activate dendritic cells and promote their migration, which contributes to hypertension pathogenesis [66]. CCR7 on dendritic cells mediates their migration to secondary lymphoid organs, where they activate T cells, which then migrate to the kidneys [66].

Renal sympathetic denervation has been shown to reduce immune cell infiltration into the kidneys and attenuate hypertension [66,185,189]. For instance, the denervation-induced decrease in macrophage infiltration in nicotine-induced hypertension was associated with lower levels of the adhesion molecule VCAM-1 and the chemoattractant MCP-1 in the kidneys [189]. In an angiotensin II-induced hypertension model, renal denervation reduced dendritic cell activation in a β2-adrenergic receptor-independent manner [66]. Consequently, renal denervation inhibited T cell activation and expansion, leading to a reduction in T cell infiltration into the kidneys [66].

Furthermore, sympathetic activation can drive immune cell infiltration into the central nervous system. Santisteban et al. showed that bone marrow-derived monocytes infiltrated the hypothalamic paraventricular nucleus in hypertensive rats, where they differentiated into microglial cells [190]. Inhibition of macrophage infiltration by minocycline attenuated hypertension [190]. Additionally, Ahmari et al. [186] found that central administration of angiotensin II in rats increased sympathetic activation of the paraventricular nucleus, which preceded sympathetic activation of the bone marrow. This activation enhanced the production of immune cells (e.g., T cells and macrophages) and promoted their migration into the circulation, a process that was inhibited by surgical sympathetic denervation [186]. Ultimately, infiltration of CD4^+^ T cells into the paraventricular nucleus marked the establishment of hypertension in these rats [186].

### 9.3. Sympathetic Activation and Immune Cell Trafficking in Cardiac Fibrosis, Hypertrophy, and Arrhythmia

**Figure 10:**
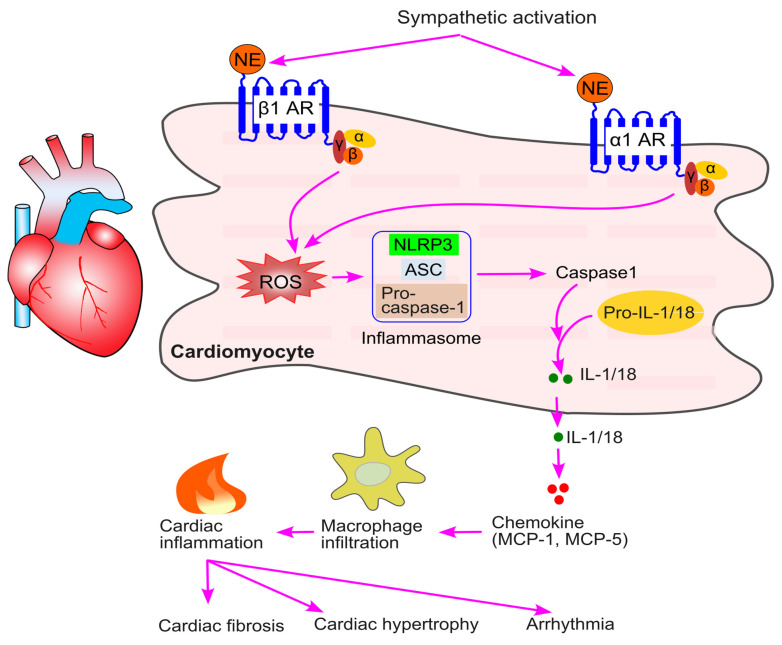
Sympathetic activation-induced immune cell infiltration in cardiac fibrosis, hypertrophy, and arrhythmia. ASC, apoptosis-associated speck-like protein containing a CARD (caspase activation and recruitment domain); AR, adrenergic receptor; IL, interleukin; NLRP3, Nod-like receptor protein 3; MCP, monocyte chemoattractant protein; ROS, reactive oxygen species.

Activation of β1-adrenergic receptors on cardiomyocytes leads to an increase in reactive oxygen species (ROS) production, which subsequently activates NLRP3 (Nod-like receptor protein 3) inflammasomes [80,85]. NLRP3 inflammasomes consist of three key components, NLRP3, ASC (apoptosis-associated speck-like protein containing a CARD), and precursor caspase-1, and they play a critical role in driving sterile inflammation across various pathologies [191,192,193]. NLRP3 activates caspase-1, which cleaves pro-IL-1 and pro-IL-18 into their active forms, IL-1 and IL-18, respectively [80,85]. These active cytokines then stimulate the production of chemokines such as MCP-1 and MCP-5 [78,194], which promote macrophage infiltration and inflammation in the heart [78,195] (Figure 10).

β1 and α1-adrenergic receptors are central to sympathetic activation-induced macrophage infiltration into cardiac tissue [196]. Pre-treatment of neonatal mouse cardiomyocytes with bisoprolol (a selective β1-adrenergic receptor blocker), but not ICI 118,551 (a selective β2-adrenergic receptor blocker), prevented isoproterenol-induced ROS production and inflammasome activation. In addition, activation of α1-adrenergic receptors also stimulates ROS formation [197] and NLRP3 inflammasome activity, leading to increased IL-18, MCP-1, MCP-5, and, consequently, macrophage infiltration into the heart [80]. Notably, physical exercise has been shown to inhibit ROS production and reduce β-adrenergic receptor activation-induced cardiac inflammation [195]. Treatment with β1-adrenergic receptor antagonists, such as propranolol [78], and α1-adrenergic receptor antagonists, such as prazosin [80], prevented sympathetic stress-induced macrophage infiltration into the heart. Xiao et al. reported that β1-adrenergic receptor activation did not affect the infiltration of T cells, B cells, or neutrophils into the heart [78].

Increased inflammation can promote myofibroblast formation and collagen deposition [198,199], ultimately leading to cardiac fibrosis [200]. Infiltrated macrophages in the heart can release proinflammatory cytokines, including tumor necrosis factor-α (TNF-α), IL-1β, and IL-6, which contribute to cardiac hypertrophy [201]. Thus, sympathetic activation-induced cardiac inflammation can eventually lead to both cardiac fibrosis and hypertrophy. Supporting this, Castoldi et al. reported that rats with angiotensin II infusion-induced cardiac fibrosis and hypertrophy exhibited higher sympathetic activity and increased infiltration of monocytes and macrophages into the heart [202]. Inhibition of sympathetic activity with empagliflozin reduced monocyte and macrophage infiltration, as well as cardiac fibrosis and hypertrophy [202]. Similarly, Higashikuni et al. found that in mice with transverse aortic constriction-induced cardiac fibrosis and hypertrophy, treatment with the β1-adrenergic receptor blocker bisoprolol inhibited macrophage infiltration and attenuated cardiac fibrosis and hypertrophy [85]. Sympathetic activation can promote cardiac fibrosis through additional mechanisms. For instance, treatment with β-adrenergic receptor blockers has been shown to inhibit the transition of human cardiac progenitor cells into a fibrotic phenotype [82,89]. This suggests that sympathetic activation may stimulate the production of cardiac fibroblasts, thereby contributing to the development of cardiac fibrosis.

Macrophages secrete numerous proinflammatory cytokines, including IL-1β, IL-6, and TNF-α, which can regulate cardiac sympathetic activity, create proarrhythmic substrates, and directly affect myocardial electrophysiology [203,204], potentially leading to arrhythmias. As a result, β-adrenergic receptor blockers are commonly used in clinical practice to treat arrhythmias [203].

### 9.4. Sympathetic Activation and Immune Cell Trafficking in Myocardial Infarction

Myocardial infarction induces sympathetic activation in both humans [205,206] and animal models [207,208]. This increase in sympathetic drive is associated with enhanced infiltration of macrophages [207,208,209] and neutrophils [207]. Studies have shown that sympathetic inhibition, through treatments such as gefapixant [207] and sinapic acid [208], reduced macrophage and neutrophil infiltration into the heart in rat models of myocardial infarction [207,208]. These interventions [207,208], along with renal denervation [210], have been demonstrated to improve cardiac function and reduce cardiac fibrosis following myocardial infarction.

**Figure 11:**
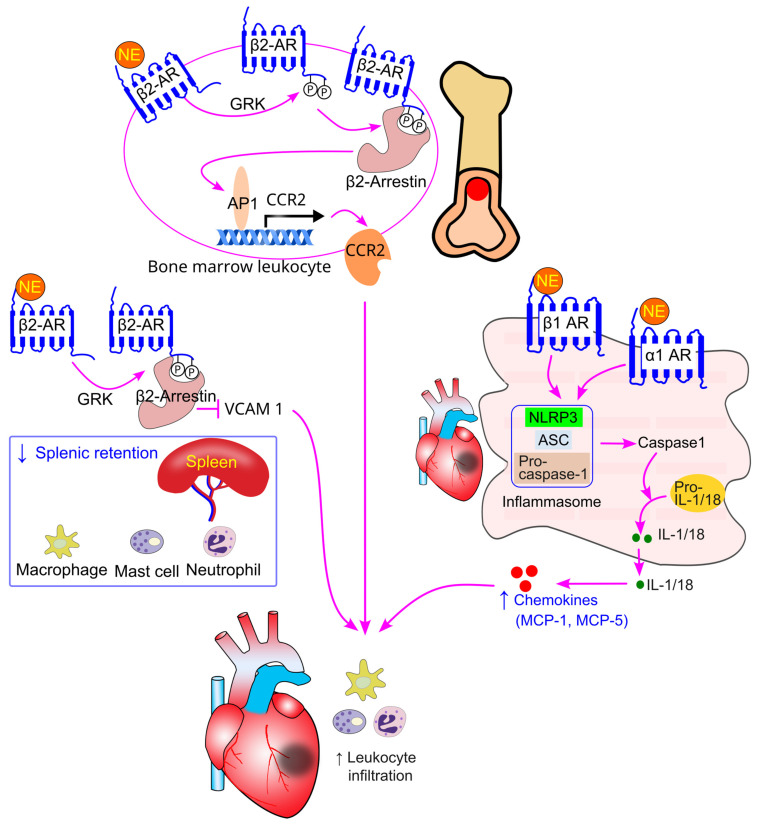
Sympathetic activation-induced immune cell infiltration in myocardial infarction. During myocardial infarction, the sympathetic nervous system becomes activated. This sympathetic activation leads to reduced splenic retention of leukocytes, increased egress of bone marrow cells, and enhanced production of chemokines (molecules that induce cell migration) in cardiac tissue. ↑, increase; ↓, decrease; AP1, activator protein-1; AR, adrenergic receptor; ASC, apoptosis-associated speck-like protein containing a CARD (caspase activation and recruitment domain); CCR2, C-C chemokine receptor 2; GRK, G-protein-coupled receptor kinase; IL, interleukin; MCP, monocyte chemoattractant protein; NLRP3, Nod-like receptor protein 3.

The sympathetic activation-induced increase in macrophage and neutrophil infiltration into cardiac tissue during myocardial infarction is partially mediated by NLRP3 activation [207], which leads to increased chemokine production, such as MCP-1, via β1 and α1-adrenergic receptor activation in cardiomyocytes (Figure 11). Targeting NLRP3 is considered a promising therapeutic strategy for preventing myocardial infarction [191]. Furthermore, β-adrenergic receptor blockers have been shown to reduce infarct size [211,212], particularly in patients with reduced ejection fraction [212,213].

In a mouse model of myocardial infarction, β2-adrenergic receptor inhibition increased spleen VCAM-1 expression [86]. Mice with β2-adrenergic receptor deficiency in bone marrow cells exhibited enhanced splenic retention of immune cells (monocytes, macrophages, mast cells, and neutrophils, but not eosinophils or T cells) after myocardial infarction [87]. These findings suggest that activation of β2-adrenergic receptors on immune cells reduces their retention in the spleen, promoting their release into circulation (Figure 11).

Moreover, in mice with myocardial infarction, β2-adrenergic receptor inhibition decreased CCR2 expression on bone marrow cells [86], thereby reducing leukocyte egress from the bone marrow and infiltration into cardiac tissue [75]. This suggests that the egress of bone marrow leukocytes plays a critical role in the sympathetic activation-induced increase in leukocyte infiltration into the heart during myocardial infarction (Figure 11).

The β2-adrenergic receptor appears to be the key receptor involved in leukocyte infiltration into the heart following myocardial infarction. Inhibition of β2-adrenergic receptors, but not β1-adrenergic receptors, reduced cardiac infiltration of monocytes, macrophages, mast cells, and neutrophils [86]. Similarly, bone marrow-specific β2-adrenergic receptor deficiency in mice significantly reduced the infiltration of monocytes, macrophages, mast cells, and neutrophils (but not eosinophils or T cells) into the heart after myocardial infarction [75,87].

It is important to note, however, that inhibition of leukocyte infiltration into the heart through β2-adrenergic receptor inhibition [86] or genetic deletion in bone marrow cells [87] led to increased mortality in mice after myocardial infarction. Notably, mice with a genetic deletion of β2-adrenergic receptors in bone marrow cells exhibited 100% mortality due to cardiac rupture following myocardial infarction [87]. These results suggest that a certain level of immune cell infiltration into the heart is essential for initiating the repair response after myocardial infarction.

### 9.5. Sympathetic Activation and Immune Cell Trafficking in Heart Failure

Heart failure occurs when the heart is unable to pump blood as efficiently as it should [214]. It can result from any condition that impairs the ventricular function, whether by hindering the heart’s ability to fill or eject blood [215]. The four most common causes of heart failure are ischemic heart disease, chronic obstructive pulmonary disease, hypertensive heart disease, and rheumatic heart disease [215].

Sympathetic activation is a key compensatory mechanism in heart failure, working to maintain adequate cardiac output [216]. However, prolonged sympathetic activation can lead to detrimental cardiac remodeling, worsening contractile function and contributing to the progression of heart failure, as well as increasing the risk of fatal events [216]. Since sympathetic activation has been shown to promote macrophage infiltration into the heart [80,85,196], β-adrenergic receptor blockers have been demonstrated to improve survival rates in heart failure patients [217,218].

von Haehling et al. found that patients with heart failure had higher circulating neutrophil counts and lower lymphocyte counts (including T and B lymphocytes) compared to healthy controls [219]. Additionally, treatment with β-adrenergic receptor blockers partially corrected these imbalances in neutrophil and lymphocyte numbers, suggesting that sympathetic activation plays a role in the observed neutrophilia and lymphocytopenia in heart failure patients.

### 9.6. Sympathetic Activation and Immune Cell Trafficking in Stroke

Stroke is a leading global cause of death, accounting for approximately 10% of all fatalities worldwide [220]. In both stroke patients and animal models, the sympathetic nervous system is activated, as evidenced by increased plasma norepinephrine levels [221,222,223,224]. Inflammation and infections are well-established risk factors for ischemic stroke [167,225,226]. The heightened sympathetic activity observed in stroke is linked to an increased risk of infections [227] and systemic immunosuppression [90], suggesting a significant neuroimmune interaction during stroke [228]. Inhibition of β-adrenergic signaling has been shown to reduce the incidence of poststroke infections in both mice [88,90] and humans [229], although some conflicting reports exist [230].

Invariant natural killer T (iNKT) cells, primarily found in the liver and spleen, are emerging as a critical immune population for regulating immune responses [231]. iNKT cells are essential for the activation of CD4^+^ and CD8^+^ T cells following stroke in mice [90]. Wong et al. [90] demonstrated that β-adrenergic receptor-mediated stationariness of iNKT cells plays a key role in stroke-induced immunosuppression. After stroke or norepinephrine administration, iNKT cell mobilization was significantly impaired, whereas iNKT cell activation helped prevent infections post-stroke. The administration of the non-specific β-adrenergic receptor blocker propranolol reversed the stroke-induced immobility of iNKT cells, thereby preventing stroke-induced lung infections and mortality [90].

The bone marrow also responds to stroke-induced sympathetic activation. Wang et al. [232] reported that stroke led to increased sympathetic activation in the bone marrow of mice, which in turn elevated regulatory T cell (Treg) numbers. This process was mediated by β2 and β3 adrenergic receptors [232]. β2-adrenergic receptor activation boosted prostaglandin E2 levels in the bone marrow, which increased receptor activator of NF-κB ligand (RANKL) expression and promoted Treg production. In addition, β3-adrenergic receptor activation decreased stromal cell-derived factor-1 (SDF-1) levels in the bone marrow, further promoting Treg production [232]. Furthermore, β3-adrenergic receptor activation facilitates the mobilization of Tregs into peripheral blood; treatment with a β3-adrenergic receptor antagonist reduced the percentage of Tregs in peripheral blood following stroke [232].

Additionally, stroke may impair neutrophil migration. Nicholls et al. found that bone marrow-derived neutrophils from stroke mice exhibited reduced migration toward chemoattractants, which was consistent with in vitro findings showing impaired neutrophil migration in the presence of higher concentrations of norepinephrine [1,133,135].

Stem cell therapy offers potential for treating stroke patients by repairing stroke-related brain damage, as stem cells can migrate to the affected brain regions and generate the necessary cells for recovery [233,234]. Evidence suggests that sympathetic nerve activity is crucial for stem cell proliferation [17], egress [76], and migration [128]. Courties et al. demonstrated that hematopoietic stem cell proliferation in the bone marrow increased following stroke, but this increase was blocked by β3 receptor genetic deletion [17]. Similarly, Spiegel et al. found that norepinephrine enhanced stem cell migration; treating CD34^+^ stem and progenitor cells with norepinephrine (1 μM) before intravenous injection into sublethally irradiated immune-deficient mice promoted their engraftment into the bone marrow [128]. Moreover, Katayama et al. showed that sympathetic nerve activity mediates granulocyte colony-stimulating factor-induced egress of hematopoietic stem and progenitor cells from the bone marrow into the circulation [76]. Pharmacological inhibition of sympathetic activity using 6-hydroxydopamine or propranolol reduced this egress [76].

## 10. Sympathetic Activation and Immune Cell Trafficking in Cancer

Cancer is one of the leading causes of death globally, responsible for nearly 10 million deaths annually [235,236]. The most prevalent types of cancer include breast, lung, colorectal, and prostate cancers [235]. Inflammation plays a crucial role in the development of cancer and is considered one of its defining characteristics [237].

Epidemiological studies have shown that the use of β-adrenergic receptor antagonists prior to cancer diagnosis is associated with slower disease progression in cancer patients [238]. Additionally, stress has been found to accelerate cancer progression in various animal models [239,240,241,242]. In many of these models, stress-induced cancer progression could be inhibited by β-adrenergic receptor antagonists and mimicked by pharmacologic β-adrenergic receptor agonists [238]. These findings suggest that sympathetic activation plays a role in tumorigenesis, and that inhibiting sympathetic activity may offer a therapeutic strategy for treating cancer [243,244].

**Figure 12:**
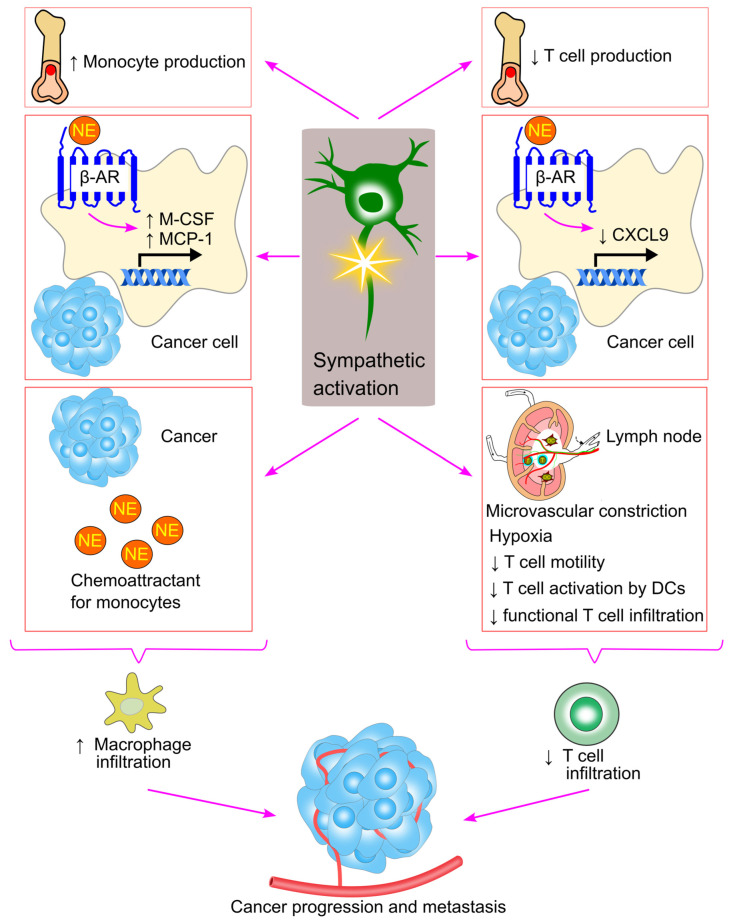
Sympathetic activation-induced immune cell infiltration in cancer. Sympathetic activation leads to enhanced monocyte/macrophage infiltration into tumor tissue by stimulating their production in the bone marrow, elevating the levels of monocyte chemoattractants (such as M-CSF and MCP-1) secreted by cancer cells, and raising norepinephrine concentrations within the tumor, which acts as a chemoattractant. In contrast, sympathetic activation reduces T cell infiltration by decreasing their production in the bone marrow, lowering the levels of T cell chemoattractant (CXCL9) produced by the cancer cells, and impairing T cell motility within the lymph node through microvascular restriction-induced hypoxia. Together, these alterations in immune cell dynamics contribute to cancer progression and metastasis. ↑, increase; ↓, decrease; AR, adrenergic receptor; CXCL9, C-X-C motif chemokine ligand 9; DC, dendritic cell; MCP-1, monocyte chemoattractant protein-1; M-CSF, macrophage colony-stimulating factor; NE, norepinephrine.

Macrophages are key players in mediating inflammation, modulating the tumor microenvironment, and promoting metastasis [239]. Macrophage infiltration into tumors can facilitate cancer progression and worsen survival outcomes for cancer patients [240,241,242]. β-adrenergic signaling has been shown to significantly enhance macrophage recruitment into the tumor by stimulating the production of chemotactic factors, such as macrophage colony-stimulating factor (M-CSF) and MCP-1, by tumor cells [79,240] (Figure 12).

Sloan et al. reported that restraint stress increased macrophage infiltration into primary breast tumor and enhanced metastasis in mice [79]. This effect was inhibited by the β-adrenergic receptor antagonist propranolol [79,245], while β-adrenergic receptor agonist isoproterenol promoted macrophage infiltration and metastasis [79]. Mechanistically, stress increased the production of M-CSF, a key macrophage chemoattractant. Inhibition of M-CSF with the pharmacological agent GW2580 blocked stress-induced macrophage infiltration into tumors and metastasis [79].

Armaiz-Pena et al. further reported that MCP-1 could facilitate stress-induced macrophage infiltration into tumors [240]. In vitro, norepinephrine increased MCP-1 secretion by tumor cells through the β2-adrenergic receptor/cAMP/PKA signaling pathway [240]. Similarly, increasing sympathetic activity through restraint stress in tumor-bearing mice boosted MCP-1 production by tumor cells, which in turn induced monocyte and macrophage recruitment into the tumor and promoted tumor growth [240]. Silencing MCP-1 expression in tumor cells inhibited macrophage infiltration and tumor growth [240], highlighting the importance of MCP-1 production in driving both macrophage infiltration and tumor progression.

β-adrenergic signaling enhances the production of precursor monocytes in the bone marrow, facilitating their formation and subsequent recruitment to the tumor microenvironment, where they differentiate into macrophages [142,239]. Furthermore, norepinephrine acts as a chemoattractant for monocytes [126,127]. As a result, heightened sympathetic activity within the tumor tissue may drive increased monocyte infiltration, owing to elevated norepinephrine levels.

Tumor infiltration by T cells is generally associated with better prognostic outcomes in many cancers [246,247,248,249]. However, sympathetic activity has been shown to inhibit T cell motility and migration, thereby promoting tumor growth [74,77]. Deletion of CD8^+^ T cells has been shown to abolish the anti-tumor effects of propranolol treatment [243].

Devi et al. reported that sympathetic activation impaired T cell motility within lymph nodes [74]. Norepinephrine activated adrenergic receptors, causing microvascular constriction in the lymph nodes, which led to hypoxia. Hypoxia raised intracellular Ca^2+^ concentrations in lymphocytes, ultimately arresting their motility within the lymph nodes [74]. This may prevent lymphocytes from encountering antigen-presenting dendritic cells, thus reducing immune responses against cancer. Consistent with this, the β2-adrenergic receptor agonist salmeterol inhibited the activation of CD8^+^ T cells in a melanoma model [74]. Similarly, Bucsek et al. found that sympathetic inhibition via propranolol treatment increased the infiltration of effector CD8^+^ T cells (expressing T-bet and interferon-γ) into tumor tissues and inhibited tumor growth [243].

Sympathetic activation can also inhibit T cell migration into cancerous tissues, promoting tumor growth. For example, norepinephrine reduced the number of CD8^+^ T cells in the cancer tissue of mice with lung adenocarcinoma [77]. Norepinephrine decreased the secretion of CXCL9 (an inducer of CD8^+^ T cell infiltration [250]) by tumor cells through the β-adrenergic receptor/WNT7A/β-catenin signaling pathway [77], thereby reducing CD8^+^ T cell infiltration. Consistently, Wrobel et al. showed that sympathetic inhibition with propranolol increased CD8^+^ T cell infiltration into melanoma tissues and suppressed tumor growth [245] (Figure 12).

Furthermore, stress has been shown to reduce the number of lymphoid progenitors in the bone marrow [142], suggesting that sympathetic activation may impair T cell production, potentially leading to reduced T cell infiltration in tumor tissues.

While sympathetic activation generally promotes cancer progression and metastasis, some studies have suggested that it may have a beneficial effect in certain cancer models. For instance, Steinberger et al. reported that restraint stress reduced macrophage infiltration and suppressed melanoma tumor growth in a mouse model [251]. However, these findings contradict other studies [245,252], which showed that sympathetic inhibition via propranolol decreased melanoma growth. These varying results highlight the complexity of sympathetic activation in cancer progression and underscore the need for further research to better understand the role of sympathetic signaling in tumor biology.

## 11. Concluding Remarks

**Figure 13:**
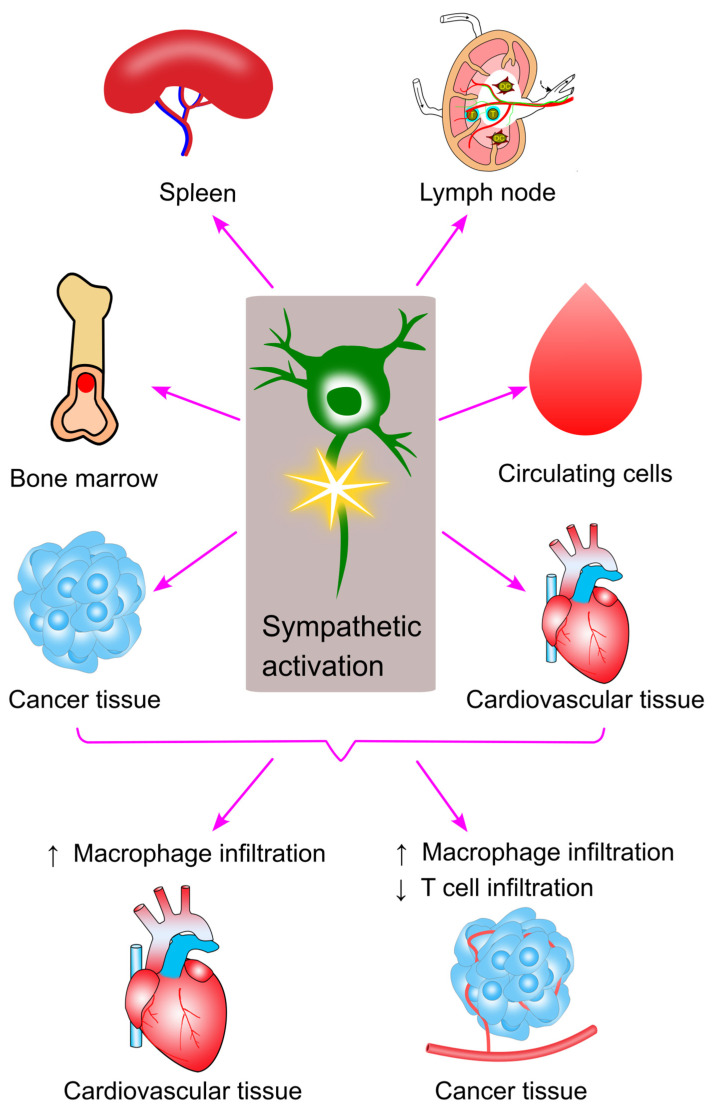
Summary. Sympathetic activation influences leukocytes in the bone marrow, spleen, lymph nodes, and circulation, regulating their migration. Additionally, it interacts with cells in cardiovascular and cancer tissues, modulating the production of chemoattractants. Overall, sympathetic activation promotes the migration and infiltration of myeloid cells—especially macrophages—into target tissues, while suppressing the infiltration of T lymphocytes. ↑, increase; ↓, decrease.

This review examined the interactions between sympathetic activation and immune cell trafficking. Current evidence suggests that sympathetic activation enhances the migration and infiltration of myeloid cells into target tissues, while inhibiting the infiltration of lymphocytes (Figure 13).

Sympathetic activation promotes inflammation by increasing the infiltration of myeloid immune cells, particularly macrophages, into affected tissues. This is observed in conditions such as cardiovascular diseases and cancer. The increased macrophage infiltration may result from (1) heightened bone marrow production and egress, (2) increased release from the spleen into circulation, and (3) elevated release of chemoattractants from the target tissue. Consequently, targeting sympathetic inhibition may provide a promising therapeutic strategy for treating both cardiovascular disease and cancer.

Furthermore, sympathetic activation can hinder T lymphocyte infiltration into cancerous tissues. This could be due to (1) reduced bone marrow production of T cells, (2) decreased motility of T cells in the lymph nodes, limiting their activation, or (3) reduced chemoattractant release from the tumor. T cell infiltration into tumors is a favorable prognostic factor in many cancers [246,247,248,249], suggesting that sympathetic activation may contribute to tumor growth by restricting T cell migration. Therefore, inhibiting sympathetic activation could offer protection against cancer by enhancing T cell infiltration and reducing macrophage accumulation in the tumor microenvironment.

Several important questions remain for future research. For instance, how does chronic sympathetic activation alter the expression of cell surface adhesion molecules and chemoattractant receptors on immune cells [139,253]? Immune cells express multiple types of adrenergic receptors—how do these different receptors interact to regulate immune cell trafficking? Sympathetic activation may also alter adrenergic receptor expression profiles. For example, stress increased β-adrenergic receptor expression in T cells, with a particularly pronounced increase in the β3-adrenergic receptor [42]. What are the implications of these changes for immune cell function and the effectiveness of sympathetic inhibition therapies? Do β3-adrenergic receptor antagonists have a role in the management of cardiovascular diseases and cancer? Additionally, since certain levels of immune cells and inflammation are essential for normal tissue repair, it is important to investigate whether the timing of sympathetic inhibition therapy, particularly after events like myocardial infarction or stroke, influences recovery outcomes. It is also important to explore the role of tertiary lymphoid organs in immune cell infiltration within cardiovascular and cancerous tissues following sympathetic activation.

## Figures and Tables

**Table 1:** Expression of adrenergic receptors on key immune cells.

Cell Type	α1 AR	α2 AR	β1 AR	β2 AR	β3 AR	Reference
T cells	√	√		√	√	[42,46,47,48,49,50,51]
CD4^+^ T cells	√	√		√		[46,47,48,49,50,51]
CD8^+^ T cells	√	√	√	√		[52,53,54,55]
B cells	√			√	√	[42,51,56,57,58,59]
Macrophages	√	√	√	√	√	[25,40,50,52,60,61,62]
Monocytes	√	√	√	√		[41,50,57,60,63]
Dendritic cells	√	√	√	√		[52,64,65,66]
Neutrophils	√	√	√	√	√	[1,41,50,67,68,69]
NK cells	√	√		√		[52,53,70,71,72,73]

AR, adrenergic receptor; NK, natural killer.

**Table 2:** The commonly used agonists and antagonists of adrenergic receptors.

Drugs	α1 AR	α2 AR	β1 AR	β2 AR	Reference
Agonists					
Phenylephrine	√				[74]
Clonidine		√			[74]
Dobutamine			√		[74]
Salbutamol				√	[75]
Salmeterol				√	[74]
Formoterol				√	[74]
Clenbuterol				√	[76]
Isoproterenol			√	√	[74,77,78,79]
Antagonists					
Prazosin	√				[80]
Phentolamine	√				[74,81]
Butoxamine		√			[82]
Atenolol			√		[77]
Bisoprolol			√		[83,84,85]
Metoprolol			√		[77,86,87,88]
Nebivolol			√		[34]
ICI 118,551				√	[83,86,87]
Nipradilol			√	√	[34]
Propranolol			√	√	[74,76,78,81,84,89,90]
Carvedilol	√		√	√	[34,86]

AR, adrenergic receptor.

**Table 3:** Effect of norepinephrine on circulating lymphocyte numbers in humans.

Dose	Route	Time	Effect	Reference
7 μg/min, for 30 min	iv	During infusion Immediately after infusion	↑ Lymphocytes	[121]
N/R	im	15 min after injection	↑ Lymphocytes	[119]
0.15 μg/kg/min, for 20 min	iv	During infusion Immediately after infusion	↑ CD3^+^ T cells ↑ CD4^+^ T cells ↑ CD8^+^ T cells ↔ CD20^+^ B cells	[123]
0.15 μg/kg/min, for 20 min	iv	30 min after infusion	↔ CD3^+^ T cells ↔ CD4^+^ T cells ↔ CD8^+^ T cells ↔ CD20^+^ B cells	[123]
7 μg/min, for 30 min	iv	30 min after infusion	↔ Lymphocytes	[121]
50 ng/kg/min, for 90 min	iv	30, 60, and 90 min after injection	↔ Helper T cells ↔ Cytolytic T cells ↔ B cells	[83]
10 μg/kg	sc	5, 15, 30, 60, and 120 min after injection	↔ CD3^+^ T cells ↔ CD4^+^ T cells ↔ CD8^+^ T cells	[120]

↑, increase; ↔, unchanged; im, intramuscular; iv, intravenous; min, minutes; N/R, not reported; sc, subcutaneous.

**Table 4:** Chemotactic effect of norepinephrine on monocytes, macrophages, and stem cells.

Cell Source	NE Concentration, Molar	Chemotactic?	Reference
Monocytes, human	10^−9^–10^−11^	Yes	[126]
Macrophages, human	10^−9^–10^−11^	Yes	[126]
Macrophages, mice	1 × 10^−8^ and 6 × 10^−8^	Yes	[127]
Macrophages, mice	3 × 10^−7^ and 6 × 10^−7^	No	[127]
CD34^+^ stem and progenitor cells, human	10^−8^ or 10^−6^	Yes	[128]

NE, norepinephrine.

**Table 5:** Effect of norepinephrine on monocyte/macrophage migration.

Cell Source	[NE], Molar	Migration Inducer	Effect on Migration	Mechanism	Ref
Macrophages, mice	10^−12^	fMLP	↑	α-ARs	[130]
Macrophages, mice aged 12 and 22 w	10^−12^	fMLP	↑	N/R	[131]
Macrophages, mice	10^−5^	fMLP	↔	N/R	[130]
Macrophages, mice aged 12 and 22 w	10^−5^	fMLP	↔	N/R	[131]
Macrophages, mice aged 48 and 72 w	10^−12^	fMLP	↔	N/R	[131]
Macrophages, mice aged 72 w	10^−5^	fMLP	↓	N/R	[131]
Macrophages, rats, treated with AlCl_3_	10^−9^ and 10^−8^	fMLP	↓	β2-AR/cAMP	[62]

↑, increase; ↓, decrease; ↔, unchanged; AR, adrenergic receptor; cAMP, cyclic adenosine 3′,5′-monophosphate; fMLP, f-Met-Leu-Phe peptide; [NE], norepinephrine concentration; N/R, not reported; Ref, reference; w, weeks.

**Table 6:** Effect of norepinephrine on neutrophil migration.

Cell Source	NE Concentration	Migration Inducer	Migration Effect	Mechanism	Reference
Human	4 × 10^−7^ M	fMLP	↑	N/R	[132]
Mice	10^−7^–10^−5^ M	fMLP	↓	N/R	[1]
Human	10^−5^–10^−3^ M	BCF	↓	β AR and cAMP	[133]
Human	10^−7^ M	Serum	↓	N/R	[135]
Mice	10^−5^ M	fMLP	↓ in vivo	N/R	[1]

↑, increase; ↓, decrease; cAMP, cyclic adenosine monophosphate; AR, adrenergic receptor; BCF, Bacterial chemotactic factor; fMLP, f-Met-Leu-Phe peptide; NE, norepinephrine; N/R, not reported.

**Table 7:** Effect of norepinephrine on lymphocyte migration.

Cell Source	NE Concentration	Migration	Mechanism	Ref
Activated CD8^+^ T cells, human	10^−5^ M	↔	N/R	[54]
CD8^+^ T cells, human	10^−6^ M	↔	N/R	[72]
Naïve CD8^+^ T cells, human	10^−5^ M	↔	N/R	[77]
Lymphocytes, mice	10^−5^ M	↓	α AR and β AR	[81]
Activated CD8^+^ T cells, human	10^−5^ M	↓	N/R	[55]
CD8^+^ T cells, human	Medium from 10^−5^ M NE-treated TCs	↓	β2 AR, ↓ CXCL9 secretion by TCs	[77]
CD8^+^ T cells, tumor-bearing mice	2 mg/mg/2 days, i.p., 7 times	↓	↓ CXCL9 secretion by tumor cells, ↓ CD8^+^ T cell infiltration	[77]

↓, decrease; ↔, unchanged; AR, adrenergic receptor; CXCL, C-X-C motif chemokine ligand; i.p., intraperitoneal; NE, norepinephrine; N/R, not reported; Ref, reference; TC, tumor cell.
